# *In Vivo* Behavior of the Antibacterial Peptide Cyclo[RRRWFW], Explored Using a 3-Hydroxychromone-Derived Fluorescent Amino Acid

**DOI:** 10.3389/fchem.2021.688446

**Published:** 2021-06-28

**Authors:** Sergii Afonin, Serhii Koniev, Laetitia Préau, Masanari Takamiya, Alexander V. Strizhak, Oleg Babii, Andrii Hrebonkin, Vasyl G. Pivovarenko, Margitta Dathe, Ferdinand le Noble, Sepand Rastegar, Uwe Strähle, Anne S. Ulrich, Igor V. Komarov

**Affiliations:** ^1^ Institute of Biological Interfaces (IBG-2), Karlsruhe Institute of Technology (KIT), Karlsruhe, Germany; ^2^ Taras Shevchenko National University of Kyiv, Kyiv, Ukraine; ^3^ Enamine, Kyiv, Ukraine; ^4^ Institute of Zoology (ZOO), Karlsruhe Institute of Technology, Karlsruhe, Germany; ^5^ Institute of Biological and Chemical Systems, Biological Information Processing (IBCS-BIP), Karlsruhe Institute of Technology, Karlsruhe, Germany; ^6^ Institute of Organic Chemistry (IOC), Karlsruhe Institute of Technology, Karlsruhe, Germany; ^7^ Leibniz-Forschungsinstitut für Molekulare Pharmakologie, (FMP), Berlin, Germany; ^8^ Lumobiotics, Karlsruhe, Germany

**Keywords:** antimicrobial peptides, arginine- and tryptophan-rich (RW) peptides, cell-penetrating peptides, fluorescent amino acids, 3-hydroxychromone, fluorescent microscopy, zebrafish embryo

## Abstract

Labeling biomolecules with fluorescent labels is an established tool for structural, biochemical, and biophysical studies; however, it remains underused for small peptides. In this work, an amino acid bearing a 3-hydroxychromone fluorophore, 2-amino-3-(2-(furan-2-yl)-3-hydroxy-4-oxo-4H-chromen-6-yl)propanoic acid (FHC), was incorporated in a known hexameric antimicrobial peptide, cyclo[RRRWFW] (cWFW), in place of aromatic residues. Circular dichroism spectropolarimetry and antibacterial activity measurements demonstrated that the FHC residue perturbs the peptide structure depending on labeling position but does not modify the activity of cWFW significantly. FHC thus can be considered an adequate label for studies of the parent peptide. Several analytical and imaging techniques were used to establish the activity of the obtained labeled cWFW analogues toward animal cells and to study the behavior of the peptides in a multicellular organism. The 3-hydroxychromone fluorophore can undergo excited-state intramolecular proton transfer (ESIPT), resulting in double-band emission from its two tautomeric forms. This feature allowed us to get insights into conformational equilibria of the labeled peptides, localize the cWFW analogues in human cells (HeLa and HEK293) and zebrafish embryos, and assess the polarity of the local environment around the label by confocal fluorescence microscopy. We found that the labeled peptides efficiently penetrated cancerous cells and localized mainly in lipid-containing and/or other nonpolar subcellular compartments. In the zebrafish embryo, the peptides remained in the bloodstream upon injection into the cardinal vein, presumably adhering to lipoproteins and/or microvesicles. They did not diffuse into any tissue to a significant extent during the first 3 h after administration. This study demonstrated the utility of fluorescent labeling by double-emission labels to evaluate biologically active peptides as potential drug candidates *in vivo*.

## Introduction

**FIGURE 1:**
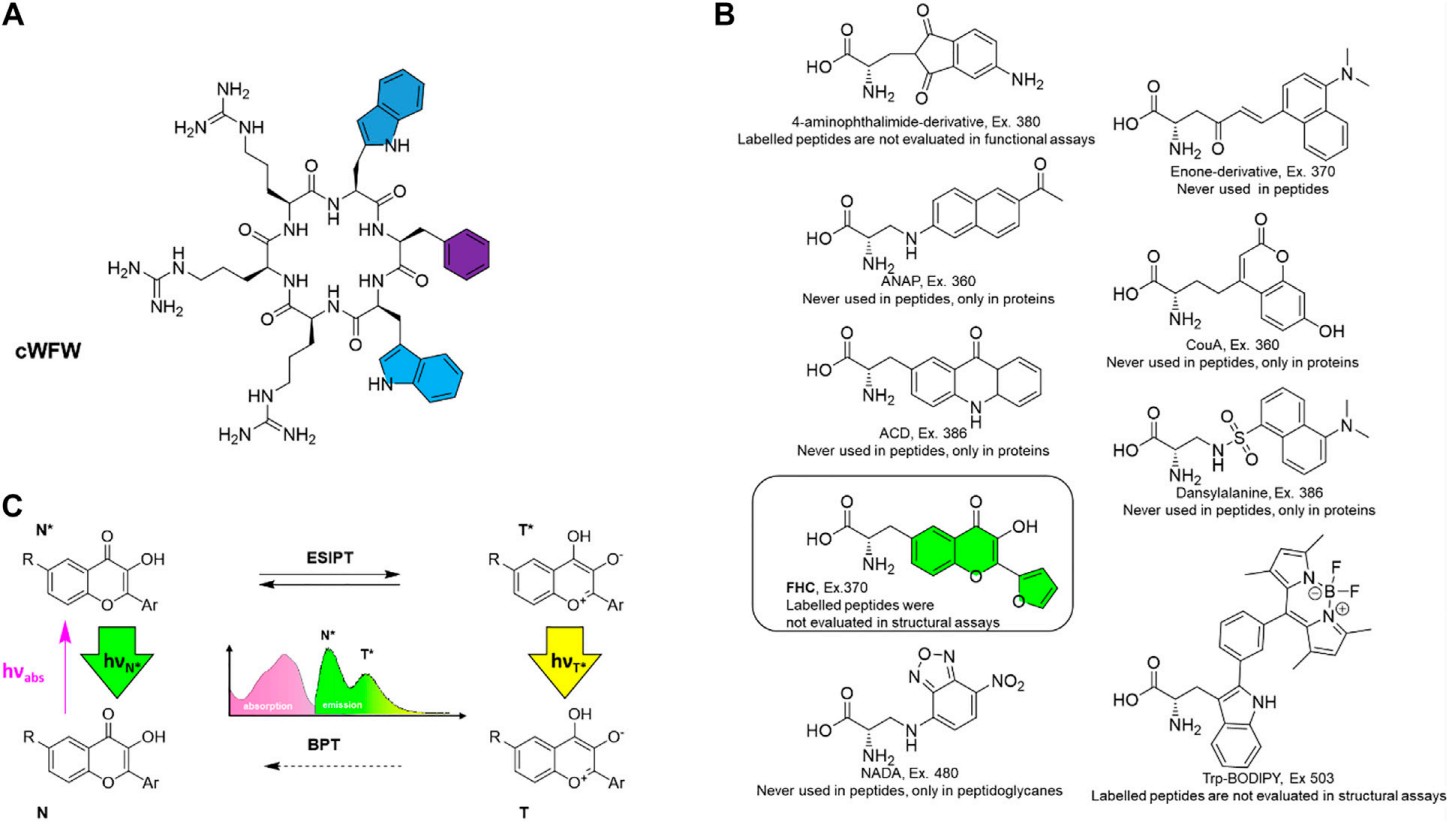
**(A)** Structure of the antimicrobial peptide cWFW with colour-highlighted aromatic residues that were individually substituted by the fluorescent label in this study; **(B)** the fluorescent amino acid FHC used as the label (framed and colored) along with potential alternatives, i.e., reported fluorescent amino acids with the excitation wavelengths higher than 360 nm. Compounds are designated according to original reports, excitation wavelengths (“Ex.”) are stated; **(C)** excited-state intramolecular proton transfer (ESIPT)-mediated fluorescence, causing dual emission and environmental sensitivity of the chromophore in 3-hydroxychromone-containing labels. N (N*) and T (T*) represent normal and tautomeric forms, respectively, in the ground (excited) state; BPT denotes back proton transfer in the ground state.

Antimicrobial peptides (AMPs) have received growing attention from the medicinal chemistry community in recent decades, as AMPs offer solutions to problems caused by acquired microbial resistance to conventional antibiotics, and they tend to demonstrate a broad spectrum (i.e., species-nonselective) antibacterial activities (Bahar and Ren, 2013; Mantravadi et al., 2019). Of particular interest is the well-documented antibiotic activity against persistent, biofilm-forming microorganisms (Berditsch et al., 2019). Among the peptides considered promising candidates for further development into antibacterial drugs, the cyclic arginine-/tryptophan-rich peptide cyclo[RRRWFW] (cWFW, Figure 1A and Table 1) stands out due to its prominent features. This peptide is one of the smallest known AMPs. It possesses enhanced biostability and remarkable selectivity against prokaryotic cells, and it effectively kills Gram-positive and Gram-negative bacteria through a unique mechanism of action that is unlikely to induce bacterial resistance (Peschel and Sahl, 2006). Several structural and mechanistic studies (Wessolowski et al., 2004; Appelt et al., 2005; Appelt et al., 2008; Junkes et al., 2008; Scheinpflug et al., 2015; Scheinpflug et al., 2017) have demonstrated that cWFW triggers a substantial reduction in cell membrane fluidity, resulting in anionic lipid clustering and membrane protein segregation. This leads to severe dysregulation of membrane functions and eventually to prokaryotic cell autolysis. The unique mode of action, high bactericidal potency, low hemolytic activity, and synthetic availability make cWFW a viable antibacterial drug candidate, justifying further studies of its behavior *in vivo*.

**TABLE 1:** Nomenclature, sequences, and characterization data of the peptides studied in this work.

Peptide	Sequence	Calc. mass^a^ [u]	Obs. Mass [u]	RT^b^ [min]
cWFW	_cyclo_[Trp^1^-Phe^2^-Trp^3^-Arg^4^-Arg^5^-Arg^6^]	988.17	988.3	9
cRW-W^1^/FHC	_cyclo_[FHC^1^-Phe^2^-Trp^3^-Arg^4^-Arg^5^-Arg^6^]	1,098.66	1,099.2	11
cRW-F^2^/FHC	_cyclo_[Trp^1^-FHC^2^-Trp^3^-Arg^4^-Arg^5^-Arg^6^]	1,137.68	1,138.0	10.5
cRW-W^3^/FHC	_cyclo_[Trp^1^-Phe^2^-FHC^3^-Arg^4^-Arg^5^-Arg^6^]	1,098.66	1,099.3	11

a Calc. and obs. masses are listed for [M + H]^+^ ions as measured in positive mode MALDI-TOF-MS.

b RT = apparent retention time as determined on C_18_ phase by HPLC with a 1% B/min gradient slope.

Amphiphilic AMPs, including cWFW, are membrane-active compounds; hence, they possess tremendous potential for activity toward not only prokaryotes but also other cell types and multicellular organisms. In particular, AMPs are now recognized as a rich source of oncolytic drug candidates or can also be used for intracellular drug delivery (Mader and Hoskin, 2006; Hoskin and Ramamoorthy, 2008; Riedl et al., 2011; Gaspar et al., 2013; Mohd et al., 2015). Despite recent success in developing drug candidates based on membrane-active AMPs (Felício et al., 2017; Ryu et al., 2021), fundamental studies on the modes and mechanisms of AMP interactions with eukaryotic cells and tissues still lag behind the studies of their antibacterial action. The studies that address the toxicity of AMPs toward different host cells and tissues are still scarce in the public domain. In most cases, such studies are limited to the estimation of erythrolysis. In this work, as one of the prime objectives, we examined human cell cytotoxicity of the cWFW and its cellular uptake in broader settings. We prepared the cWFW analogues labeled with a fluorescent amino acid residue and carried out *in vivo* observations of the analogues using not only a representative human cancer line and noncancerous cells, but also *Danio rerio* (zebrafish) embryos as a multicellular animal model organism.

Unnatural fluorescent amino acids have been successfully used for labeling large proteins and elongated polypeptides and for their consecutive observation in cells and tissues by fluorescent microscopy (Lee, 2019; Cheng et al., 2020; Laxman et al., 2021). However, in the case of short peptides which possess less than 20 amino acid residues, the applicability of this potent exploration tool is hampered by severe structural and functional perturbations often caused by the label. As previous studies have shown, small hexameric cWFW is particularly sensitive to such perturbations (Scheinpflug et al., 2013); therefore, cWFW labeling presents a considerable challenge. This sensitivity and our intention to use the labeled analogues for studies in living objects prompted us to choose the fluorescent amino acid for the labeling very carefully.

First, although many unnatural fluorescent amino acids exist and multiple biomolecular assays and *post mortem* imaging applications have been reported in the literature (Toseland, 2013; Cheng et al., 2020), they were surprisingly rarely reasonably exploited *in vivo*. The cause is that many reported fluorescent amino acids possess excitation maxima in the short-wavelength ultraviolet (UV) range, detrimental to live cells. Therefore, we upfront considered the known fluorescent amino acids with high fluorescence quantum yield and excitation maxima above 350–360 nm. We were surprised that only a few of them had been reported in peptides, and none of the peptides has been investigated simultaneously in structural and functional assays. In most reports, either the function of a fluorescent analogue has not been compared with the prototype peptide, or a structural evaluation has been omitted (Figure 1B).

Second, for our study, we decided to use 2-furanyl-3-hydroxychromone-derived amino acid FHC, as it is a double-emission label. It has been demonstrated previously as an efficient probe in characterizing peptide-DNA interactions (Strizhak et al., 2012). Labels of this type are known as 3-hydroxychromone (3HC) probes (reviewed in Klymchenko et al., 2012). They are unique among the peptide fluorescent labels as they undergo rapid excited-state intramolecular proton transfer (ESIPT, Figure 1C). Both, normal (N*) and tautomeric (T*) excited states exhibit discrete emission bands, the position and relative intensity of which are sensitive to the environment. In particular, polar and H-bonding media suppress the ESIPT, thus decreasing the relative intensity of the T* band. Therefore, double-emission labels such as the FHC-amino acid are considered superior for studies of biological systems (Pivovarenko et al., 2012). Such ratiometric fluorescent probes can be incorporated in place of natural amino acid residues to convey information about the peptide and its immediate surroundings through several channels, as opposed to single-channel intensometric probes. 3HC-derived labels have already demonstrated excellent performance in studies of AMP interactions with model lipid bilayers and cell membranes (Postupalenko et al., 2011; Postupalenko et al., 2013; Zamotaiev et al., 2014), where only a few efficient instrumental methods currently exist.

Lastly, we desired to cause minimal perturbations to cWFW upon labeling. Although several 3HC-based amino acids that are superior to FHC in their spectroscopic characteristics have been described to date (Zamotaiev et al., 2011; Postupalenko et al., 2013; Sholokh et al., 2015), we chose FHC because this molecule is the most compact and, except for the hydroxycarbonyl moiety, possesses no other reactive groups in the side-chain that could further affect the peptide structure and functions. Additionally, in comparison against other known 3HC probes, FHC residue possesses hydrophobicity coefficient LogP_C_ closest to tryptophan and phenylalanine. Therefore, we envisaged that FHC should be most appropriate to substitute aromatic residues in cWFW, highlighted by color in Figure 1A.

## Materials and Methods

### Ethical Statement

The experimental design complied with European Legislation for the Protection of Animals used for Scientific Purposes (Directive 2010/63/EU). All experiments with *Danio rerio* were performed on larvae less then 120 h postfertilization (hpf), which does not require an ethical commission approval (Afonin et al., 2020). Experiments were performed following the relevant German animal protection standards.

### Materials

All chemicals were purchased from Sigma-Aldrich, Iris Biotech or Biosolve and were used without further purification. Ultrapure deionized (MilliQ, Millipore) water was used to prepare aqueous solutions and mixtures. Solvents for peptide purification and optical spectroscopy were of HPLC grade and were degassed before use. Culture media and supplements for animal cells were from Thermo Fisher, those for bacterial culturing were from Becton, and those for zebrafish embryo handling were freshly prepared. Citrate phosphate dextrose-stabilized blood bags with erythrocyte suspensions of healthy donors for hemolysis assays were obtained from the blood bank of the Karlsruhe municipal hospital.

### Cell Cultures

*Bacillus spizizenii* (DSM 347) and *Escherichia coli* (DSM 1116) were purchased from the German Collection of Microorganisms and Cell Cultures. The bacterial strains were maintained frozen at −80°C using a bacterial Cryobank System (Mast Diagnostica). To refresh bacterial cells on single glass beads, they were grown overnight in Müller-Hinton (MH) medium, and colonies were obtained by streaking on MH agar. Then, colony cells were used to inoculate MH broth up to OD = 0.02 and grew at 37°C overnight. These overnight cultures were used to inoculate the test culture, which was grown for 3–4 h until it reached exponential growth. HeLa (ECACC 93021013) and HEK293 (ECACC 85120602) cells were purchased from American Type Culture Collection and were routinely cultured in Roswell Park Memorial Institute (RPMI) 1640 medium or Dulbecco’s modified Eagle medium (DMEM) supplemented with 10% fetal calf serum (FCS) and 1% penicillin/streptomycin, respectively. Cells were passaged every 2–3 days and used until passage 35. All animal cell types were cultured at 37 °C in a 5% CO_2_ atmosphere and tested negative for *mycoplasma*.

### Zebrafish Embryos

All experiments were performed using wild-type (strain AB) *Danio rerio*. Zebrafish housing and husbandry were performed following the recommendations by (Aleström et al., 2020). Fertilized eggs (6 hpf) were raised in 4 ml of embryonic medium (E3, 5 mM NaCl, 0.17 mM KCl, 0.33 mM CaCl_2_, 0.33 mM MgSO_4_, 0.1% methylene blue) at 28 °C until 72 hpf (6-well plate, 20 eggs/well).

### Preparation of (*S*)-2-Amino-3-(2-(Furan-2-yl)-3-Hydroxy-4-Oxo-4H-Chromen-6-yl)propanoic Acid

The synthesis of Fmoc (fluorenylmethyloxycarbonyl)-protected FHC followed procedures described in (Strizhak et al., 2012).

### Solid-Phase Peptide Synthesis

Peptides were synthesized with a BiotageSyro II automatic peptide synthesizer using standard solid-phase Fmoc protocols. The linear sequences were synthesized on a 2-chlorotrityl (2CT) resin preloaded with the first amino acid arginine (H-L-Arg (Pbf)-2CT resin). The resin (200–400 mesh) load was 0.5 mmol/g; the synthesis scale was 0.2 mmol. The sequences for the linear peptides were as follows: linearWFW: H-Arg (Pbf)-Arg (Pbf)-Arg (Pbf)-Trp (Boc)-Phe-Trp (Boc)-NH_2_, linearRW-W1/FHC: H-Arg (Pbf)-Arg (Pbf)-Arg (Pbf)-FHC(Ac)-Phe-Trp (Boc)-NH_2_, linearRW-F^2^/FHC: H-Arg (Pbf)-Arg (Pbf)-Arg (Pbf)-Trp (Boc)-FHC(Ac)-Trp (Boc)-NH_2_, and linearRW-W^3^/FHC: H-Arg (Pbf)-Arg (Pbf)-Arg (Pbf)-Trp (Boc)-Phe-FHC(Ac)-NH_2_. For standard α-amino acids, double coupling with 4 equiv was used. Fmoc-protected amino acids were activated with HBTU (2-(1*H*-benzotriazol-1-yl)-1,1,3,3-tetramethyluronium hexafluorophosphate) and HOBt (1-hydroxybenzotriazole) using *N*,*N*-diisopropylethylamine (DIPEA) in *N*-methyl pyrrolidone (NMP) as the reaction solvent. Fmoc deprotection in all cases was performed with 20% piperidine in NMP. The coupling of the fluorescent amino acid was performed manually at selected positions using 1.2 equiv. of Fmoc-FHC, activated with 1.2 equiv of PyOAP (7-azabenzotriazol-1-yloxy)tripyrrolidino-phosphonium hexafluorophosphate) and 2.4 equiv of DIPEA in *N*,*N*-dimethylmethanamide. Then, the resin was washed with DMF and acylated (0.5 ml acetic anhydride, 4 ml DMF and 1 ml DIPEA, 15 min). Following acylation, the resin was extensively washed with NMP and synthesis was continued on the peptide synthesizer. After completion of a linear sequence, the resin was washed with DCM and dried under vacuum. Linear precursors were cleaved from the resin without side-chain deprotection by a mixture (1:3, v/v) of HFIP (1,1,1,3,3,3-hexafluoropropan-2-ol) and methylene chloride (DCM). The resulting solution was filtered from the resin and dried using a rotary evaporator. The obtained oil was suspended in an acetonitrile/water mixture (1:1, v/v) and lyophilized. The cyclization step was performed in DCM (0.8 L per 0.2 mmol load) with the activating mixture of PyOAP (2 equiv) pre-dissolved in DMF, followed by the addition of DIPEA (4 equiv). The reaction mixture was stirred overnight. The solvent was removed on a rotary evaporator, and the residual material was lyophilized. The final deprotection of the cyclized peptides was performed with a deprotecting cocktail containing trifluoroacetic acid (TFA), triisopropylsilane, and water (95:2.5:2.5, v/v/v). The volatiles were removed on a rotary evaporator, and the residual oil was suspended in acetonitrile/water and lyophilized.

The peptides were purified (>95%) by reversed-phase high-performance liquid chromatography (HPLC) employing C18 stationary phase columns (Vydac) and TFA-supplemented (0.1% v/v) water/acetonitrile gradients (4% eluent B/min) without temperature control on a Jasco HPLC system equipped with a diode array detector. Analytical chromatography was performed using the same stationary/mobile phase conditions, employing temperature control (column temperature 40°C) and shallower gradients (1% eluent B/min). All peptides and individual HPLC fractions were characterized by matrix-assisted laser desorption/ionization time-of-flight mass spectrometry (MALDI-TOF MS) using a Bruker Autoflex III instrument in positive reflector detection mode and with α-cyano-4-hydroxycinnamic acid as a matrix. From pure peptide powders, stock solutions were prepared (1 mg/ml) in 30% aqueous acetonitrile and stored at −80°C. For spectroscopy, imaging and bioactivity measurements, aliquots from these solutions were taken a day before the experiment, and peptide samples containing the necessary amount of peptide dry material were obtained by lyophilization overnight.

### Circular Dichroism Spectropolarimetry

Salt-free phosphate buffer (PB, 10 mM, pH 7.4), 2,2,2-trifluoroethanol (TFE), or 10 mM aqueous sodium lauryl sulfate suspension were added to dry peptides (final concentration 100 µM), vortexed and incubated for 30 min at r.t. In the form of large unilamellar vesicles (LUVs), liposomes were prepared by conventional extrusion at a 2 mM concentration of the total lipid in PB. Extrusion was performed at 35°C through 0.1 µM polycarbonate membranes (Nucleopore) using Avanti Mini Extruder. Stable LUV suspensions were obtained either from 8:2 M mixture of 1,2-dimyristoyl-*sn*-glycero-3-phosphocholine (DMPC) with cholesterol, or from 1:1 M mixture of DMPC with 1,2-dimyristoyl-*sn*-glycero-3-phospho-(1′-*rac*-glycerol (DMPG). The former mixture mimics a typical zwitterionic “animal” membrane lipid composition; the latter represents an anionic “bacterial” membrane model counterpart. LUV suspensions were gently mixed with the 200 μM PB solutions of peptides and incubated for 30 min at 35°C to allow membrane binding above lipid mixture melting temperatures. CD spectra were measured on a J-815 Jasco spectropolarimeter. The measurements were performed in quartz glass cells (Hellma) with a 1 mm path length between 260 and 185 nm at 0.1 nm intervals. Spectra were recorded at 25°C and for LUVs at 35°C using a water-thermostatted rectangular cell holder. Three repeat scans at a scan rate of 20 nm/min, an 8 s response time and a 1 nm bandwidth were averaged for each spectrum. After subtracting the baseline (peptide-free samples), CD data were processed with Jasco Spectra Analysis software.

### Spectroscopic Measurements

Dry peptides were dissolved in ultrapure deionized water, PB, TFE, ethanol or DMF to a concentration of 8 µM. Baseline-corrected absorption spectra were recorded at r.t. in single-beam mode on a Cary 4000 UV–visible (UV-VIS) spectrophotometer (Varian) using 500 µl volume, 10 mm light-path length quartz glass cells (Hellma). Fluorescence measurements were performed immediately after UV-VIS measurements on a FluoroMax2 spectrofluorometer (HORIBA Jobin Yvon) equipped with a thermostated cell compartment without cell exchange (excitation wavelength varied; typically, 1 nm slits were used for both excitation and emission channels). The data were processed and analyzed in OriginPro 2020b.

### Bacterial Growth Inhibition Assay

The bacteriostatic activity of the peptides was characterized by determining minimal culture growth inhibitory concentrations (MICs) in planktonic cultures. Serial twofold dilutions of each peptide (in-well concentration range: 128 μg/ml to 2 mg/ml) were prepared in MH broth and aliquoted into round-bottom 96-well microtiter plates (Sarstedt). Each well was inoculated with a standardized inoculum to achieve a final test concentration of approximately 10^6^ cells/ml. The MIC was measured as the lowest concentration of the peptide that completely inhibited visible bacterial growth after incubation at 37°C for 20 h and was further confirmed by resazurin staining (addition of 20 µl of 0.2 mg/ml resazurin to each well and incubation at 37°C for an additional 2 h). The measurements were repeated twice with three replicates each time.

### Hemolysis Assay

Hemolytic activity was examined using the same serial twofold dilution concentrations as for the MIC assay. The erythrocytes were washed with isotonic Tris-HCl buffer (pH 7.6, 37°C) diluted to a 0.5% hematocrit, equal volumes of peptide Tris-HCl buffer solutions were added, and mixtures were incubated at 37°C for 30 min with gentle shaking. The tubes were centrifuged at 13,000 rpm for 10 min to pellet the cells, and the absorbance at 540 nm was recorded against a negative control (cells without peptide, accounting for autohaemolysis). The lysis percentage was calculated relative to lysis levels induced by 1% (v/v) Triton X-100. The absorbance measurements were repeated five times, and the averaged values were used.

### Cell Viability by Fluorescent Staining

A day before imaging, cells were split and transferred to 4-well borosilicate microscopy chambers (chambered cover glass from LabTek), allowed to adhere and cultured in RPMI or DMEM at 37°C, 5% CO_2_, 10% FCS, and 1% penicillin/streptomycin. For imaging, each well was washed with Leibovit’s L-15 (L-15) medium without phenol red and other supplements; 500 µl of a freshly prepared L-15 solution of peptides was applied to each well, and chambers were incubated at 37°C in a 5% CO_2_ atmosphere. Imaging was performed at 37°C without CO_2_ supplementation. To quantify cell viability (2.5 h after peptide addition), an additional 500 µl of L-15 solution of propidium iodide (PI) and Hoechst 33342 (Hoechst) was added to each well (the final concentrations were 1 μg/ml for PI and 3 μg/ml for Hoechst). After a 10 min incubation, images were acquired at 20× magnification using laser excitation at 405 and 488 nm. Three images per well at randomly chosen positions were collected. Maximum-intensity projections of the z-stacks around the highest cell density focal plane were generated and analyzed using ImageJ/Fiji software.

### Fluorescence Microscopy

For peptide imaging, cell cultures were treated, and peptides were applied as described above. In cell culture experiments, z-stack image acquisition was performed with either a Z1 Cell Observer spinning disc inverse microscope (Zeiss) or an inverse confocal laser scanning microscope SP8 (Leica Microsystems), both equipped with a 20× NA 0.75 water or 63× NA 1.4 oil immersion objective and several solid-state excitation lasers, including a 405 nm laser.

For intravital imaging, anesthetized living three dpf zebrafish embryos were embedded, with the right lateral side of the embryo facing upward, into 0.8% (w/v) low-melting agarose (Lonza) in 35 mm glass-bottom microscopy dishes (MatTek). The agarose was covered with E3 medium supplemented with 0.017% (w/v) tricaine methanesulfonate and 0.003% (w/v) 1-phenyl-2-thiourea for anesthesia and background fluorescence reduction, respectively. For injections, peptides were prepared as 100 µM solutions in PBS. Each solution was injected (ca. 8 nL) with borosilicate glass capillary tubes (∼0.6 mm diameter) into the common cardinal vein of the immobilized subjects with a gas-driven microinjector (Eppendorf Femtojet express), and dishes were transferred to the microscope where images were acquired at r.t. using an SP8 upright confocal microscope (Leica Microsystems) equipped with a tunable white light laser for double-photon excitation. Leica instruments were equipped with LAS X software for image acquisition. Maximum intensity projections were generated and analyzed using ImageJ/Fiji software.

## Results and Discussion

### Validation of the Label

An essential step of all studies involving peptide labeling is the validation of the label. It should be confirmed that the label, incorporated instead of an appropriate peptide fragment, disturbs neither the parent peptide structure nor functions significantly. For the small, constrained cWFW peptide, choosing such a non-disturbing label is a challenging task. This peptide has already been studied using intensometric fluorescent labels incorporated in place of aromatic residues (Scheinpflug et al., 2013). In most known cases, any modifications by the fluorescent probes led to drastic changes in the cWFW structure and biological activity compared to the original cWFW sequence. These data were decisive for our choice of the label, the amino acid FHC, and we first verified experimentally if our choice was justified.

To compare individual aromatic substitutions, we synthesized three cWFW analogues (Table 1), where the FHC residue substituted for W^1^, F^2^, or W^3^ in the parent peptide. Gratifyingly, *N*-Fmoc-FHC demonstrated excellent compatibility with Fmoc solid-phase peptide synthesis (SPPS) protocols.

**FIGURE 2:**
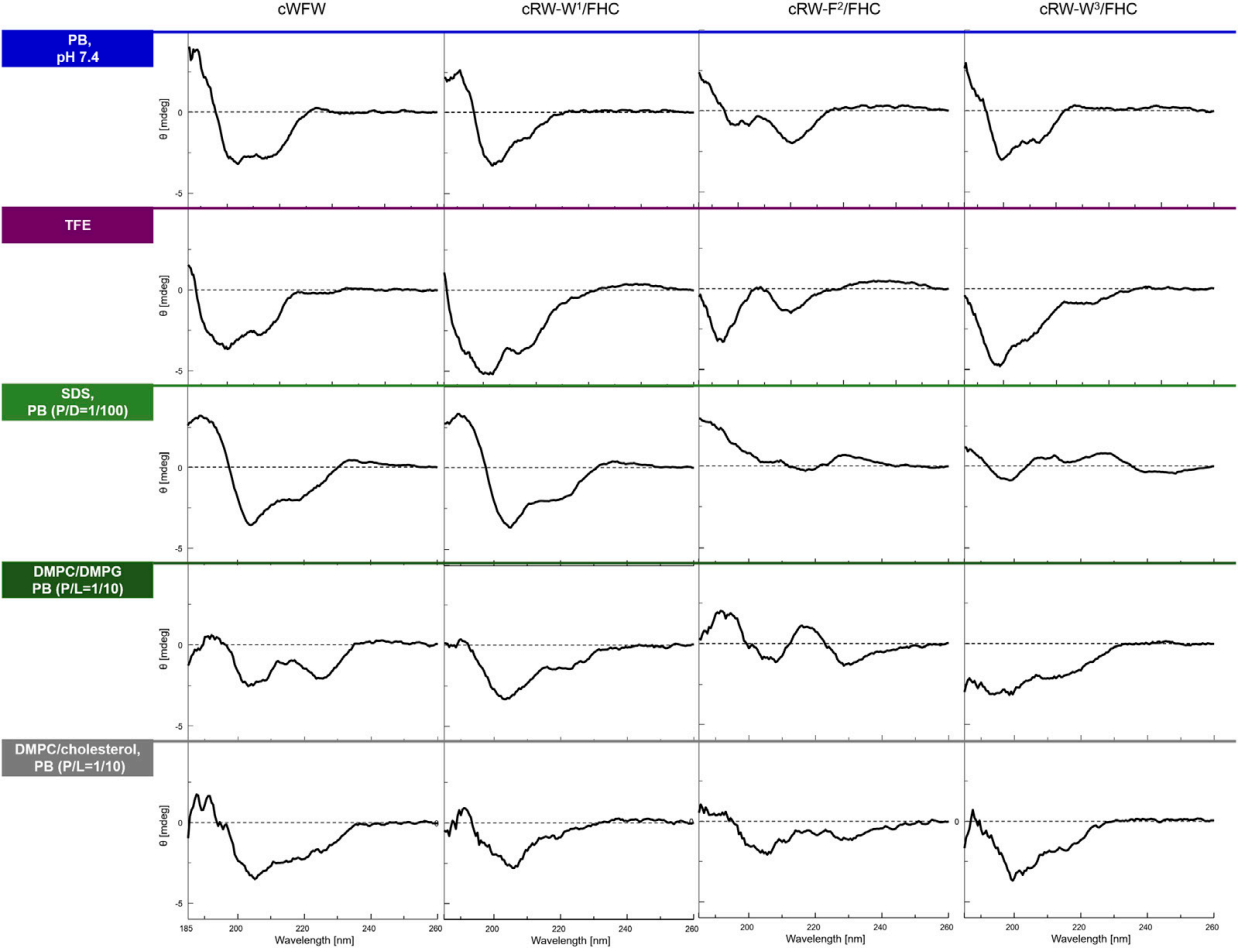
CD spectra of the peptides in various environments (top to bottom traces): in 10 mM phosphate buffer, pH 7,4, in 2,2,2-trifluoroethanol (TFE), in 10 mM sodium dodecyl sulphate (SDS) micelles at a peptide/detergent ratio (P/D) of 1/100, in the presence of large unilamellar vesicles (LUVs) modeling an “animal” (DMPC/cholesterol, 4/1) or a “bacterial” (DMPC/DMPG, 1/1) plasma membrane in terms of the lipid composition. The spectra were acquired from fresh 100 µM (or 200 µM for LUVs) peptide solutions at 25°C or 35°C (LUVs). P/L designates peptide/lipid ratio.

Conformational preferences of the three labeled peptides were evaluated in different environments using CD spectropolarimetry and were then compared with the parent peptide. We measured the CD spectra in PB (10 mM, pH 7.4, 25°C), in 100% TFE, in the presence of detergent micelles (SDS, excess), and in the presence of prokaryotic or animal membrane composition mimicking LUVs, to determine whether the environment affects the conformation of the cWFW-derived analogues and to model conditions of the following *in vivo* studies. As shown in Figure 2, all the peptides revealed CD patterns compatible with a combination of turn-like structures (Bagheri et al., 2011; Hernández et al., 2012), as would have been expected for a cyclic hexamer peptide. Since all peptides, including cWFW, exhibited different CD lineshapes in different environments, one may assume a certain degree of conformational freedom in the peptide backbone, despite its cyclic and thus backbone-constrained nature. The CD spectra revealed that the nature of the environment strongly affects conformational equilibria; in particular, the averaged conformation of each peptide was very different in aqueous and membrane-mimicking environments. We also noted that all peptides demonstrated similar overall lineshapes in each of the two isotropic solutions (Figure 2 top two rows). However, an exact match to the parent cWFW was not observed in any of the analogues for the entire conditions set. cRW-F^2^/FHC was the most deviant from cWFW under the tested conditions. In contrast, both Trp/FHC-substituted peptides appeared to be folded similarly to cWFW, and we note that both analogues fold most similarly to the parent cWFW in the presence of an “animal” cell membrane surrogate.

In the next step, we tested various biological activities of the labeled peptides, namely, their bacteriostatic, hemolytic, and cell-penetrating abilities. The latter has never been measured for a nonlabelled cWFW, whereas the first two activities had been severely compromised upon labeling, as reported in previous studies (Scheinpflug et al., 2013).

**FIGURE 3:**
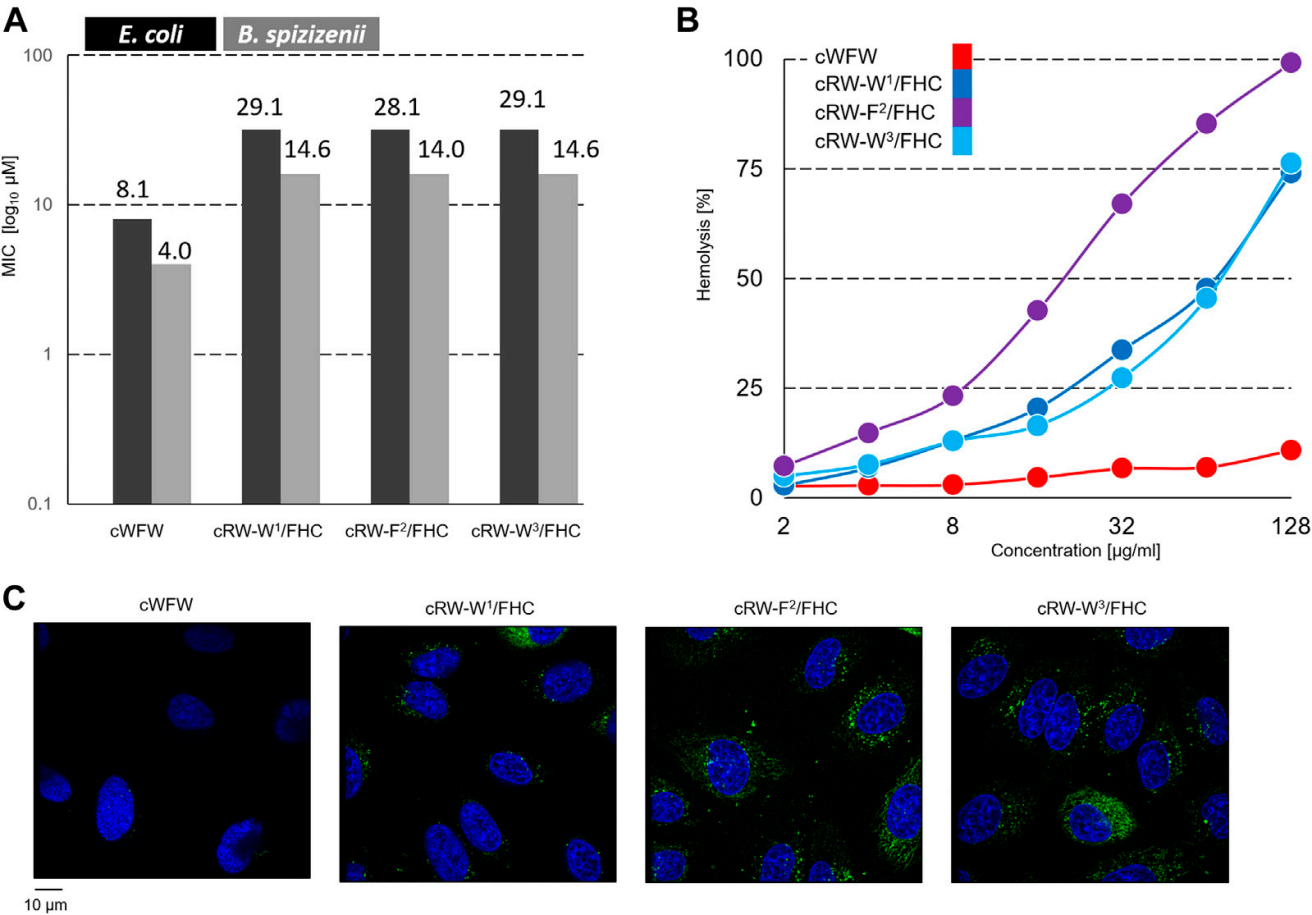
**(A)** Planktonic bacterial growth inhibition according to a broth microdilution assay. Numbers depict µM values calculated from µg/ml dilutions; **(B)** concentration-dependent hemoglobin release from human erythrocyte suspension; and **(C)** fluorescence microscopy observation of Hoechst 33342 (blue color)-co-stained Hela cells after 1 h of incubation with 30 µM FHC-labelled cWFW analogues (green color). Images are 105 × 114 µm.

We measured the minimum inhibitory concentrations (MIC values) for the peptides using two representative bacterial strains: a gram-negative *E. coli* and a gram-positive *B. spizizenii*–by a standard twofold dilution assay. The results are shown in Figure 3A. All labeled peptides demonstrated equal (within the experimental errors) bacteriostatic activity, which was still relatively high and differed from the MIC of cWFW by two dilutions. The best-performing fluorescent label used previously, nitrobenzoxadiazole-modified diaminopropionic acid, had caused a decrease in bacteriostatic activity by three to four dilutions (Scheinpflug et al., 2013). Interestingly, despite pronounced conformational differences (Figure 2), all the labeled analogues were indistinguishable in terms of their MIC values.

In contrast, the hemolysis assay revealed different activities of the labeled peptides compared to cWFW. All three labeled analogues appeared more hemolytic than the parent molecule. The peptide cRW-F^2^/FHC was the most active among the series, showing hemoglobin releasing potency compatible with the previously studied fluorescent cWFW analogues (Scheinpflug et al., 2013). The two Trp/FHC-substituted peptides revealed significantly lower hemolysis values. Hemolytic activity did not correlate with the overall hydrophobicity of the peptides (as assessed by HPLC retention time, see Table 1). In our hands, cRW-F^2^/FHC was the most hemolytic but was less polar than both Trp/FHC analogues.

Next, we incubated the fluorescent cWFW analogues with an adherent HeLa cell culture and compared the ability of the peptides to enter the cells by confocal fluorescence microscopy. As shown in Figure 3C, FHC-labeled peptides indeed entered the cells, demonstrating both cytosolic and microsomal entry. No differences were observed among the three cWFW analogues. We also noted that the overall cell entry (cell staining) was not uniform, as some cells were stained more intensely than the others.

From the studies described in this section, we concluded that FHC exerts modest perturbation of the structure and functions of the parent peptide cWFW. These perturbations are less drastic than those reported previously for this peptide; hence, our choice of the label is justified for the present study. Among three analogues, cRW-W^3^/FHC overall appeared to behave closest to the parent peptide.

### Photophysical Properties of Fluorescently Labeled Peptides

**FIGURE 4:**
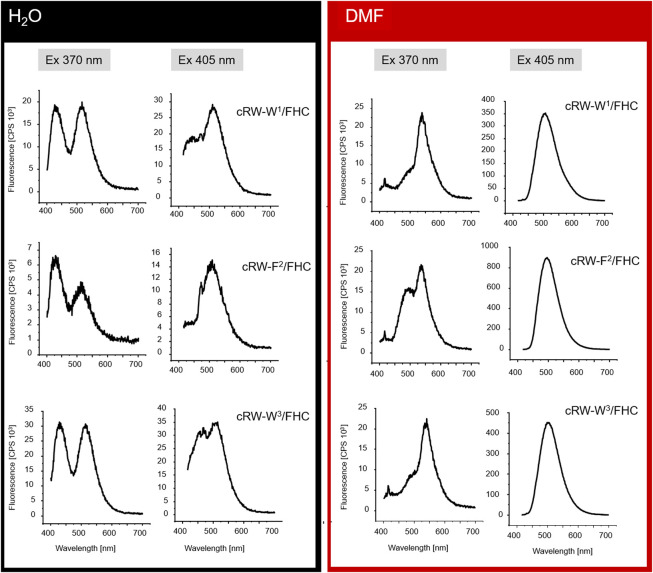
Fluorescence spectra (not normalized) from 8 µM solutions of the FHC-labeled cWFW analogues in H_2_O and DMF under different excitation wavelengths (indicated as “Ex 370 nm” and “Ex 405 nm”).

To explore the environmental sensitivity of the fluorescent spectral parameters of the FHC-labelled peptides, we collected their fluorescence spectra (under excitation at 365 nm) in PB, TFE, and ethanol (data not shown). These spectra confirmed the dependence of the position and relative intensity of the T*/N* emission bands on the solvent polarity and H-donor ability. However, many confocal microscopes are not equipped with UV lasers exciting at wavelengths lower than 405 nm; hence, we tried to observe the fluorescence of the same samples using excitation at 405 nm. Surprisingly, we observed a significant deviation of the emission spectra from those obtained under 365 nm excitation. Therefore, in the next set of experiments, we systematically addressed the spectral characteristics of the peptides. First, we compared the fluorescence spectra of all the labeled peptides at 370 and 405 nm excitation in solvents of different polarities, water and DMF (Figure 4).

Under excitation at 370 nm in water, the T*/N* band intensity ratio was influenced by the position of the label in the peptide sequence. The two Trp/FHC-substituted analogues revealed comparable ratios in their emission spectra. In contrast, the spectrum of cRW-F^2^/FHC appeared to possess a lower T*/N* band ratio, correlating with the outstanding conformational behavior and higher hemolysis rate observed for this peptide. Together with the lower fluorescence intensity, this spectral change may reflect a more hydrated local environment of the F^2^/FHC residue in this analogue, suggesting a much higher number of water molecules around the residue in this position (FHC in the labeled analogue or Phe in the wild-type peptide). It is well known that in aqueous surroundings, both the quantum yields and the T*/N ratios of 3HC dyes diminish (Klymchenko et al., 2007; Klymchenko et al., 2008); therefore, it is plausible that the residue at this position is more exposed on average to bulk water than its immediate neighboring residues.

In the spectra excited at 405 nm in water, we observed the opposite scenario: the double-emission spectrum of cRW-F^2^/FHC appeared to possess the highest T*/N* band ratio, suggesting the lowest number of water molecules surrounding the fluorophore. It appears, therefore, that the conformational equilibrium of cRW-F^2^/FHC in water should include at least two dominant conformations: in the former, the FHC residue is exposed to the bulk water, and in the latter, it is partially shielded by the other amino acid residues.

Next, we note that for all analogues, the overall fluorescence intensity remained similar in both solvents at 370 nm excitation. However, the signal was an order of magnitude brighter in DMF than in water under 405 nm excitation. Taking into account the generally higher quantum yields of the FHC fluorophore in DMF (Klymchenko and Demchenko, 2004; Klymchenko et al., 2008) and the higher basicity of DMF compared to water (Reichardt and Welton, 2019), the observed intensity difference can be explained by an alternate distribution of the ground-state tautomers of FHC (Figure 1C) in the two solvents or by anion formation of FHC. It has been previously shown for the parent 2-furylchromones that the anionic form of the dye could be present in measurable quantities even at pH 7.4 (Klymchenko and Demchenko, 2004). The species were assigned by absorption maxima of 413 nm for the anionic forms and 358 nm for the neutral forms in that model study. In our experiments under 405 nm excitation, an increase in fluorescence intensity in DMF was observed for all peptides, allowing the assumption that these spectra correspond to the emission of the much brighter anionic form (however, see the discussion below).

**FIGURE 5:**
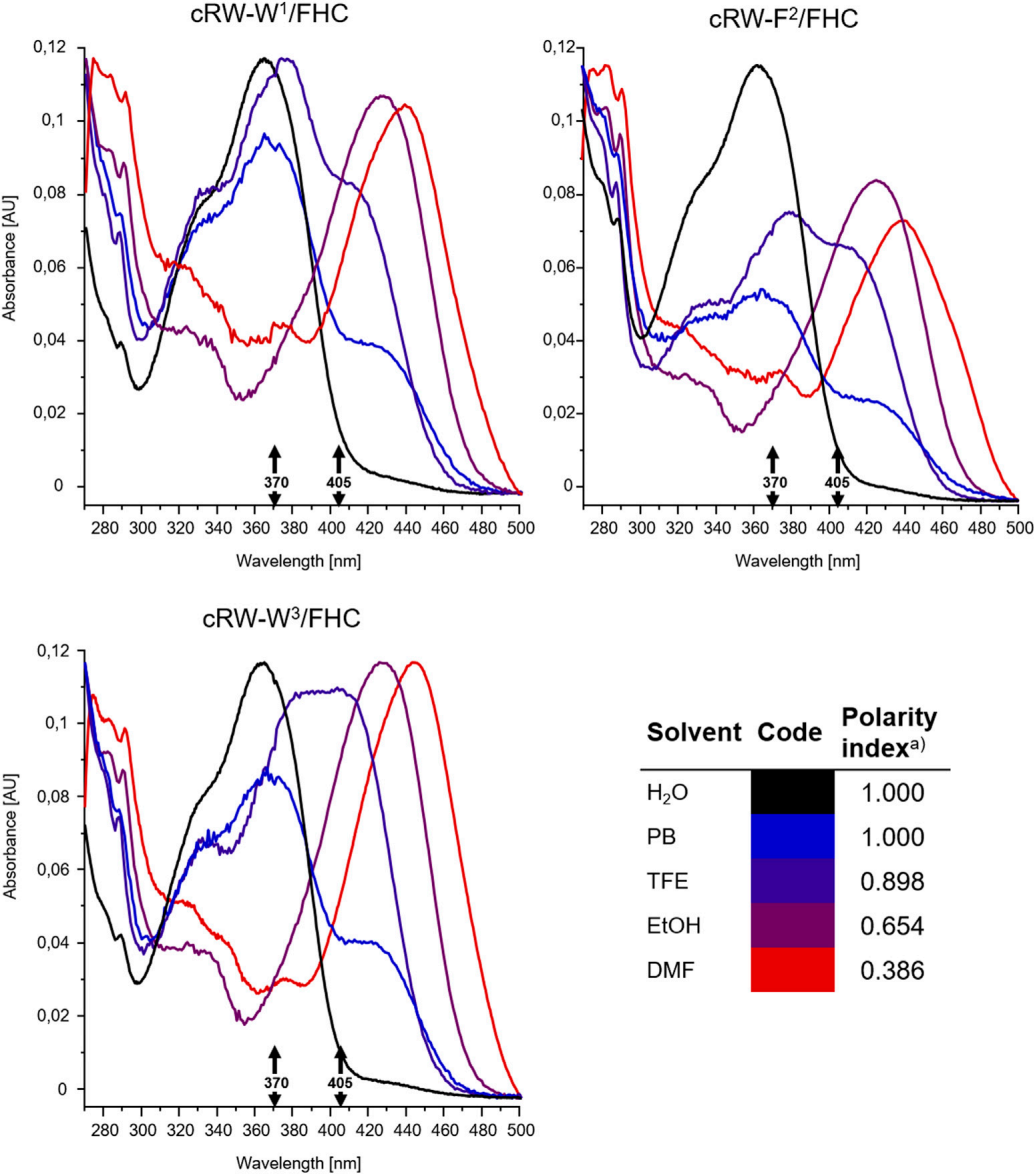
Intensity-normalized absorbance spectra from 8 µM solutions of the FHC-labelled cWFW analogues in solvents of different polarities, colored according to the polarity index (Reichardt and Welton, 2019, insert, “a)”). Black arrows mark excitation wavelengths used for fluorescence measurements and microscopy (370 and 405 nm).

**FIGURE 6:**
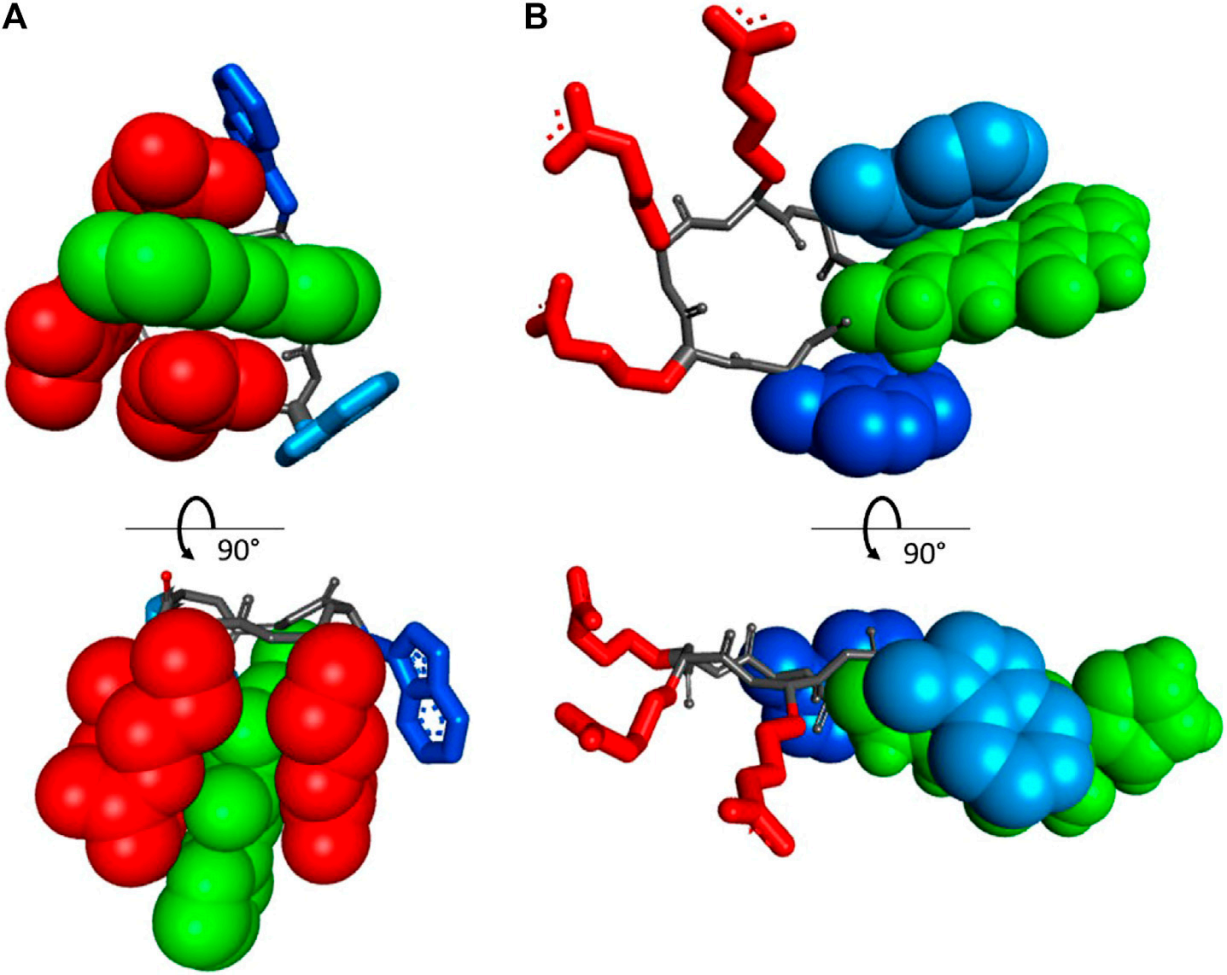
Orthogonal projections of the two energetically preferred conformations *in vacuo* of the peptide cRW-F^2^/FHC, according to an MM+ molecular modeling run: **(A)** “closed” state, where all three arginyls interact with the FHC side-chain, and **(B)** “open” state, where the arginyl cluster and the aromatic side-chains are well separated. The FHC residue is in green and CPK. Trp^1^ and Trp^3^ are in dark and light blue, respectively. The residues interacting with FHC are shown in CPK rendering mode.

The excitation wavelength-dependent change in the fluorescence of our peptides prompted us to examine their absorption properties in more detail. We recorded UV-VIS spectra for the peptides in solvents of different polarities (Figure 5). Surprisingly, we detected significant solvatochromism in the absorption spectra of our 3HC-containing peptides. A notable observation was the ∼90 nm bathochromic shift of the absorbance maxima upon changing the solvent from H_2_O to DMF. When water and DMF were the solvents, the spectral difference correlated with the emission changes. Additionally, this might be explained as a result of the increased pH at the location of the FHC chromophore, leading to an increased population of the ground state anionic form. A consecutive increase in the solvent basicity explains spectral changes in the order: water > PB (Klymchenko and Demchenko, 2004) > ethanol > DMF. However, the extreme shift of the absorbance maximum of the anionic form to 455 nm in DMF and an intermediate spectral shape in TFE (which is more acidic than water (Sierra et al., 2005; Reichardt and Welton, 2019) cannot be explained by the changes in polarity or basicity of the solvent alone. In DMF, in particular, both forms of the FHC chromophore, neutral and anionic, were reported to absorb at much lower wavelengths (Klymchenko and Demchenko, 2004; Klymchenko et al., 2007). We suggest that this abnormal redshift of the absorption maxima may result from intramolecular side-chain/side-chain interactions. Guanidinium groups of the cationic arginyls can be in close proximity to the FHC residue, promoting the anionic form and modulating the fluorescence quantum yield. Similar stabilization of the chromophore ground state accompanied by a substantial increase in the fluorescence quantum yield was reported earlier for complexes of a 3HC with the tetracharged anion of adenosine-5′-phosphate (Pivovarenko et al., 2017). Molecular modeling corroborates this suggestion: the constrained cWFW backbone can still allow Arg-FHC interactions (Figure 6).

Overall, we suggest that in aqueous solutions, the cWFW analogues (and possibly cWFW) exist in more than one conformation, including a structure with aromatic side-chains residing near the charged residues (“closed” state, Figure 6A). Peptide molecules in this conformation should be those that absorb light at 390–470 nm and are adequately excited at 405 nm. This pool of molecules gives an intense emission peak in the fluorescence spectrum at 490–520 nm. The alternative conformational set, where the aromatic side-chains may participate in various mutual π-π interactions, pointing away from the charged groups of the arginine residues (“open” state, Figure 6B), should be responsible for the double-emission spectrum, with maxima at approximately 430 and 530 nm. These suggestions do not contradict the results of the NMR structural studies of cWFW in different environments (Appelt et al., 2008) nor our CD data.

Changing of the environment from aqueous to less polar can promote shifting the conformational equilibria toward conformations with effective WFW-RRR side-chain interactions, as seen from the UV-VIS absorption spectra and from the presence of the emission peak at 490 nm in the fluorescence spectra of all analogues under 370 nm wavelength excitation. Therefore, this peak is assigned to the Arg-stabilized anionic form of the FHC fluorophore in the “closed” state.

Collectively, excitation of the FHC-labelled cWFW analogues at 370 nm provides a fluorescence response mainly from the “open” state conformations since the fluorescence intensity from the “closed” states is weak (∼10–20% of total intensity). Excitation at 405 nm will mainly sample the molecules in “closed”-like states.

### Cell Entry and Cytotoxicity of cWFW Analogues Studied in Human Cells

Next, we continued to study the peptide-cell interactions by co-incubating the labeled cWFW analogues with adherent cells, comparing different cell lines (HeLa cancer line vs HEK293 noncancer line), and varying peptide concentrations (1–60 µM), and incubation times (5 min–2.5 h). To assess the polarity of the media surrounding the FHC residue, we recorded the emissions separately within a spectral range corresponding to the N* (415–480 nm) and T* (490–555 nm) bands, as well as the total 416–600 nm signal. Due to the constructional peculiarities of the confocal microscope used, the excitation in these experiments was performed using the 405 nm laser.

**FIGURE 7:**
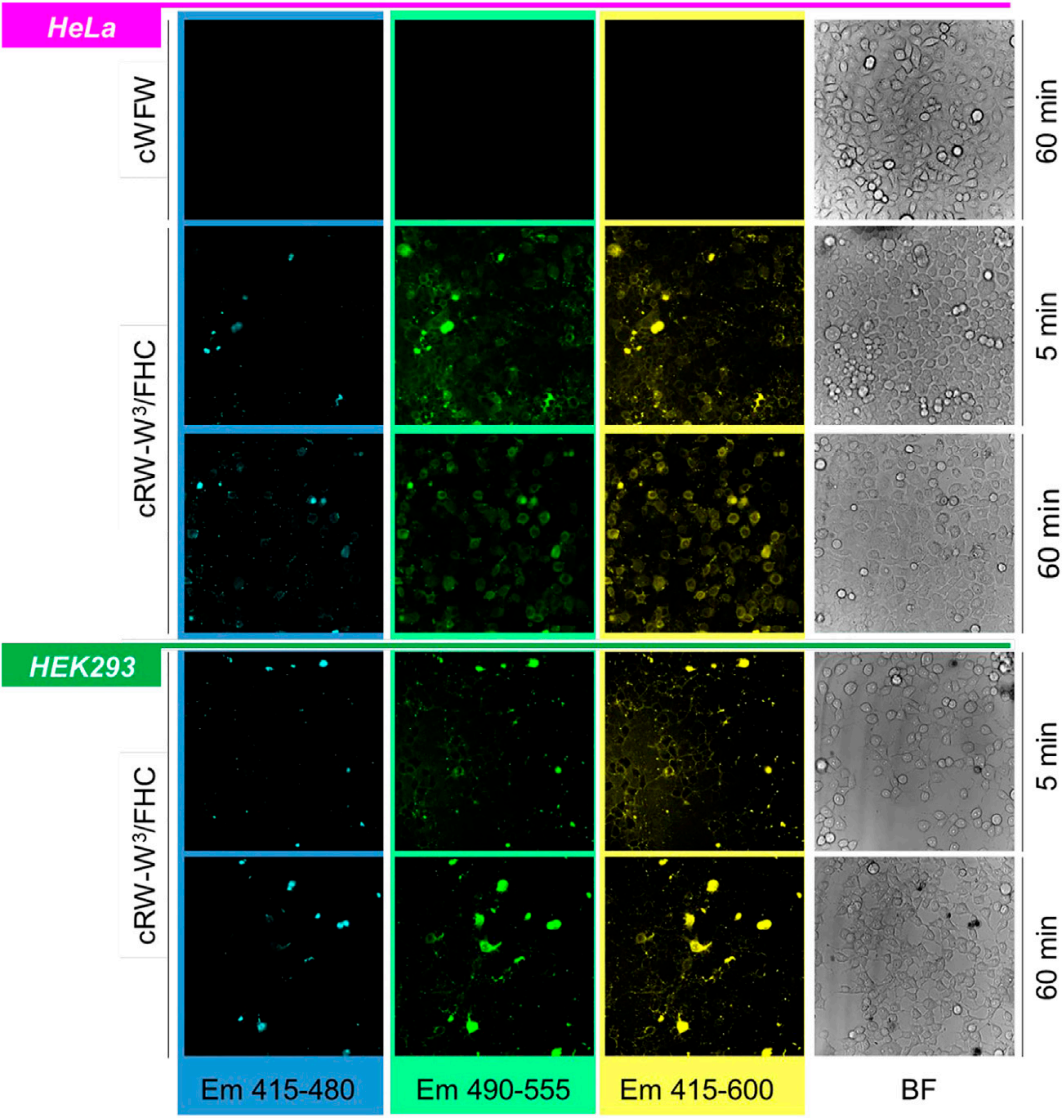
Band-filtered confocal fluorescence images of adherent HeLa and HEK293 cell cultures incubated with 30 µM cRW-W^3^/FHC for 5 and 60 min (indicated). Spectral ranges for collecting emission data are shown at the bottom of the image stacks; BF = bright-field images.

**FIGURE 8:**
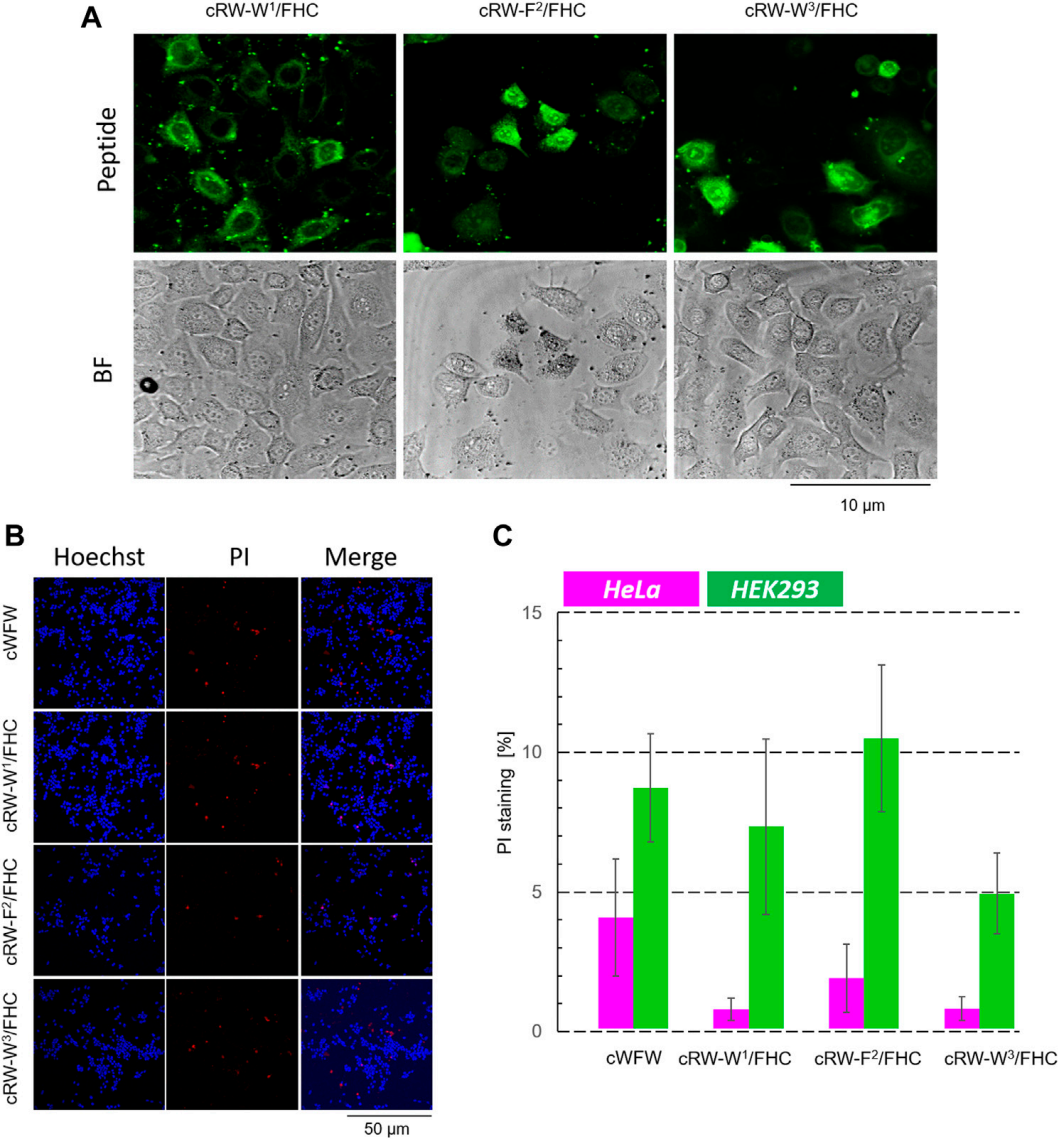
Cytotoxicity evaluation of cWFW and its fluorescent analogues by fluorescence microscopy. **(A)** First, at higher magnification (63× objective), the cells were imaged to monitor cell entry of the peptides; HeLa cells with 20 µM peptide imaging after 1 h of incubation are shown for illustration. **(B)** After imaging (2.5 h after peptide addition), Hoechst/PI co-staining is applied and imaged at 20× magnification, exemplified here by experiments with HEK293 cells; **(C)** Total/dead cell counting results for all peptides at 20 µM concentration co-incubated with both cell types.

As shown in the representative images (Figures 7, 8A), detection within the three different spectral ranges at the chosen magnification revealed the same signal patterns, which differed only in absolute intensity. This fact should correspond to a low N*/high T*-emission, characteristic of a decreased polarity of the FHC-surroundings. In a living animal cell, the membranes, lipid droplets, and supramolecular protein complexes in the cytoplasm may provide such environments.

Immediately after peptide addition (∼5 min incubation), a strong signal associated with the plasma membranes was visible for both types of cells. Following 1 h of incubation, the images changed. In HeLa cells, the cell boundaries remained barely visible, while much stronger cytosolic and microsome-associated signals emerged. We noted that the cell nuclei remained unstained by the labeled peptides under all conditions studied. This behavior in HeLa cells was observed in the entire concentration range of 1, 20, 30, and 60 µM for all peptides, independent of the FHC-fluorophore position. In contrast, HEK293 cells retained the fluorescence mainly in the plasma membranes even after prolonged (2.5 h) incubation with the peptides. We conclude from these observations that cWFW analogues efficiently entered cancer cells but not the noncancerous undifferentiated cells.

The occurrence of bright, uniformly stained cells with unusual morphology that we observed upon incubation of both, HeLa and HEK293 cells, with the labeled peptides could correspond to necrotic cells. As can be assessed from the hemolytic activity data (Figure 3), the Trp/FHC-substituted peptides cause the death of >25% erythrocytes at a 30-µM concentration, and even more (>50%) in the case of the Phe/FHC-analogue. We used a method based on fluorescence microscopy to quantify the cytotoxic potency against HeLa and HEK293 cells under imaging conditions. After cell-entry imaging, i.e., after 2.5 h co-incubation, we incubated the cells with Hoechst and PI (propidium iodide). Imaging the Hoechst (blue) and PI (red) staining revealed, respectively, the total cell number and the membrane-damaged, apparently, necrotic cells. Representative images and the results of these experiments are illustrated and summarized in Figure 8.

Contrary to the haemolysis results, we observed that the labeled cWFW analogues demonstrated relatively low toxicity (<5% at 20 µM) against HeLa cells. In the case of HEK293 cells, the cytotoxicity was slightly higher and comparable to the cWFW toxicity. The peptides killed 5–10% of the HEK293 cells at 20 μM, but this activity was still lower than the hemolytic activity (Figure 3).

### The Biodistribution of Peptides *in Vivo*

**FIGURE 9:**
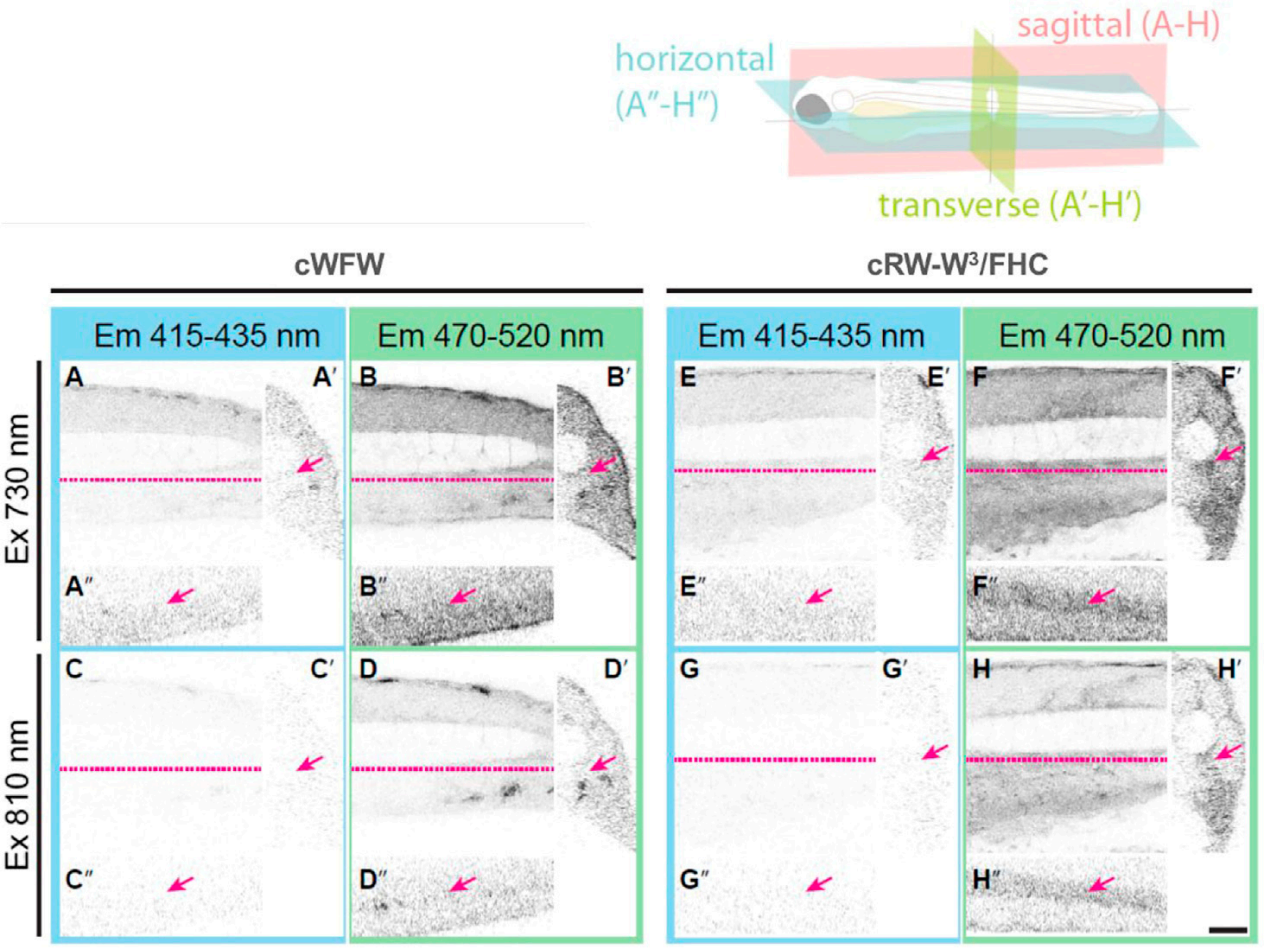
Orthogonal section views from four dpf zebrafish embryos injected with either cWFW in PBS **(A–D)** or cRW-W^3^/FHC peptide **(E–H)**. The trunk region was imaged by detecting emitted fluorescence light in a range of either 415–435 nm **(A, C, E, G)** or 470–520 nm **(B, D, F, H)** after sequential two-photon excitation at 730 nm **(A, B, E, F)** and 810 nm **(C, D, G, H)**. Sagittal sections **(A–H)**, transverse sections **(A′–H′)** and horizontal sections **(A′′–H′′)** are shown. Dashed lines (magenta) in sagittal views indicate the position where horizontal views were made. Arrows indicate the caudal artery, where cRW-W^3^/FHC and other peptides were mainly found to be localized. Scale bar: 50 µm.

After confirming the low toxicity of the fluorescently labeled peptides against animal cells, we administered the compounds to four dpf zebrafish embryos via intravenous injection. A total of 8–10 nl of 100 µM peptide solution in PBS was applied to every agarose-immobilized subject, followed by confocal imaging after 30 min of incubation. The biodistribution of each peptide was analyzed in XYZ dimensions by detecting fluorescent signals emitted in a range of either 415–435 nm or 470–520 nm followed by sequential two-photon excitation at 730 and 810 nm, roughly corresponding to 365 and 405 nm single-photon excitation, respectively (representative images are shown in Figure 9).

With the applied procedure, we did not detect any signs of toxicity during 3 h of imaging. There was also no pronounced difference between the peptides. For all of the peptides, we observed fluorescence intensity associated only within the blood vessels. Interestingly, assessment of the T^*^/N^*^ band intensity ratio, similar to the cell experiments described above, revealed a local hydrophobic environment around the FHC-label. At the same time, we see that the peptides remained in the blood. This observation suggests that the cWFW analogues were not dissolved in the plasma. Instead, they must have associated most likely with blood lipoproteins, apoptotic bodies, exosomes and/or microvesicles (Hyenne et al., 2019), while not binding significantly to the cellular fraction in the blood.

## Conclusion

In summary, we have validated a known 3-hydroxychromone-derived dual-fluorescent amino acid FHC as a “good” peptide label for *in vitro* and *in vivo* studies of peptides in bio-objects, useful even in the highly constrained context of an ultrashort cyclic hexameric peptide. This label can deliver valuable information on conformational behavior, localization, polarity of the environment, basicity, and the H-bond donating/accepting ability of the molecules in close vicinity to the fluorophore through various spectroscopic and fluorescence imaging techniques. The use of the FHC label in the antimicrobial peptide cWFW revealed a cell-penetrating activity of this peptide toward the cancerous cell line HeLa. This functionality correlates well with its observed localization in cellular membranes or other cellular components with low polarity. The same label also helped to localize the peptide in a living model organism, the *D. rerio* embryo, and characterize its microenvironment. The cytotoxic effects of cWFW and its fluorescent analogues toward cancerous cells were not high enough to consider these peptides as possible anticancer agents. However, given the great potential of cWFW as an antimicrobial drug candidate, our observations of the interactions of cWFW analogues with animal cells and the multicellular organism described above provided meaningful data. In particular, the confinement of the labeled cWFW analogues to the bloodstream and their inefficient penetration into tissues, as observed in the experiments with *D. rerio*, suggest the need for further optimization of formulations for this compound to improve its *in vivo* biodistribution. Further experiments using herein designed FHC-labelled analogues and possibly other HC-based derivatives might also clarify the molecular details of the cWFW peptide conformational equilibria in various native and model environments and provide valuable insights into the mechanisms of antibacterial and other bioactivities.

## Data Availability

The original contributions presented in the study are included in the article/supplementary material, further inquiries can be directed to the corresponding authors.
